# Endowing protein language models with structural knowledge

**DOI:** 10.1093/bioinformatics/btaf582

**Published:** 2025-10-24

**Authors:** Philip Hartout, Dexiong Chen, Paolo Pellizzoni, Carlos Oliver, Karsten Borgwardt

**Affiliations:** Department of Machine Learning and Systems Biology, Max Planck Institute of Biochemistry, Bavaria, Martinsried 82152, Germany; Department of Machine Learning and Systems Biology, Max Planck Institute of Biochemistry, Bavaria, Martinsried 82152, Germany; Department of Machine Learning and Systems Biology, Max Planck Institute of Biochemistry, Bavaria, Martinsried 82152, Germany; Department of Machine Learning and Systems Biology, Max Planck Institute of Biochemistry, Bavaria, Martinsried 82152, Germany; Department of Machine Learning and Systems Biology, Max Planck Institute of Biochemistry, Bavaria, Martinsried 82152, Germany

## Abstract

**Motivation:**

Protein language models (PLMs) have transformed protein research by learning rich representations from sequence data alone, yet they largely ignore the wealth of structural information now available through advances in structure prediction. Current methods that incorporate structural data often require substantial computational resources and complex architectures, limiting their practical adoption. We present a novel joint sequence and structure embedding method that achieves computational and parameter efficiency while maintaining high performance. Our approach introduces a lightweight integration framework that combines pretrained sequence transformers’ self-attention with specialized structural adapters, enabling seamless incorporation of structural knowledge into existing PLMs through these enhanced self-attention mechanisms.

**Results:**

The method demonstrates remarkable efficiency, requiring only modest pretraining on 542K protein structures, three orders of magnitude less than the data used to train PLMs, using standard masked language modeling objectives. Despite this lightweight approach, our joint embeddings consistently outperform sequence-only models like ESM-2 while achieving comparable results to more complex structure-based methods that use significantly more parameters and computational resources. This work establishes a new paradigm for protein representation learning that balances performance with practical constraints. By providing computationally efficient joint sequence-structure embeddings, we offer the scientific community an accessible tool that captures both sequential and structural protein information without the computational overhead typically associated with structure-aware models.

**Availability and implementation:**

code and links to checkpoints are available at https://github.com/BorgwardtLab/PST.

## 1 Introduction

Proteins are fundamental building blocks of life, supporting diverse biological functions through three-dimensional folds whose geometry and physico-chemical characteristics dictate specific biological roles. Disruptions in these processes can lead to diseases, making the relationship between protein sequence, structure, and function crucial for therapeutic innovations (Bhardwaj *et al.* 2022, Ren *et al.* 2023).

Protein language models (PLMs) have transformed protein research by learning rich representations from sequence data alone (Rives *et al.* 2021). Through self-supervised pretraining on hundreds of millions of sequences, PLMs capture secondary and tertiary structures, enable atomic-level structure prediction (Lin *et al.* 2023), and demonstrate proficiency in predicting protein functions, including mutation effects (Meier *et al.* 2021), metal ion binding (Hu *et al.* 2022), and antibiotic resistance (Hu *et al.* 2022).

Despite these advances, PLMs largely ignore structural information now available through advances like AlphaFold (Jumper *et al.* 2021), which have revolutionized structural database availability (Varadi *et al.* 2022). Current structure-incorporating methods often require substantial computational resources and complex architectures, limiting practical adoption. Early attempts (Gligorijević *et al.* 2021, Wang *et al.* 2022) introduced complex pretraining pipelines that rarely surpass PLM performance. Recent studies (Zhang *et al.* 2023a, 2023c) enhance PLMs by overlaying structure encoders, yielding parameter-inefficient models requiring expensive task-specific fine-tuning. Inverse folding approaches (Ingraham *et al.* 2019, Jing *et al.* 2020, Gao *et al.* 2022, Dauparas *et al.* 2022) deduce sequence from structure alone but exclude sequence data, not fully exploiting structure-sequence synergies.

We present a novel joint sequence-structure embedding method achieving computational and parameter efficiency while building on the base PLM performance. Drawing on graph transformer advances (Chen *et al.* 2022), we introduce a lightweight framework combining pretrained sequence transformers’ self-attention with specialized structural adapters, enabling efficient incorporation of structural inductive biases through enhanced self-attention mechanisms, which can be applied to any PLM. Our approach enhances ESM-2 (Lin *et al.* 2023) by fusing structure extractor modules within its self-attention blocks.

The method demonstrates remarkable efficiency, requiring only modest pretraining on 542K protein structures, approximately three orders of magnitude less data than PLMs—using standard masked language modeling. Notably, *refining only structure extractors while keeping the backbone transformer frozen yields substantial improvements*, addressing parameter efficiency concerns. The resulting protein structure transformer (PST) can extract representations directly or be fine-tuned for specific tasks.

Our joint embeddings consistently outperform sequence-only models while achieving comparable results to complex structure-based methods using significantly fewer parameters and less computation. Unlike prior structure-based models (Zhang *et al.* 2023a, 2023c) requiring expensive fine-tuning, PST representations serve broad purposes with simple linear or multilayer perceptron layers. Our method compares favorably on Enzyme and Gene Ontology predictions (Gligorijević *et al.* 2021) and ProteinShake benchmarks (Kucera *et al.* 2023).

Our findings underscore the potential of integrating structural information into PLMs, establishing a new paradigm for protein representation learning that balances performance with practical constraints.

## 2 Related work

Protein representation models can be categorized by their primary input modality into sequence-based, structure-based, and hybrid models.

### 2.1 Sequence-based models

Transformer models have demonstrated strong performance in protein function prediction when self-supervised pretrained on large protein sequence datasets (Rao *et al.* 2019, Hu *et al.* 2022, Elnaggar *et al.* 2021, Rives *et al.* 2021, Lin *et al.* 2023). Notable examples include ESM-1b (Rives *et al.* 2021) and ESM-2 (Lin *et al.* 2023), which have achieved strong results on various downstream tasks (Meier *et al.* 2021, Notin *et al.* 2022, Kroll *et al.* 2023). See Appendix A.1, available as supplementary data at *Bioinformatics* online for additional sequence-based approaches.

### 2.2 Structure-based models

Given the evolutionary conservation of protein structures and their direct influence on functionality (Illergård *et al.* 2009), structure-based models often provide more accurate predictions (Townshend *et al.* 2020). Graph Neural Networks (GNNs) have emerged as particularly effective, with models like GVP (Jing *et al.* 2020) and GearNet (Zhang *et al.* 2023a) leveraging geometric structure representations. The proliferation of protein folding models (Jumper *et al.* 2021) and databases like AlphaFold (Varadi *et al.* 2022) has further enabled structure-based approaches (additional structure-based methods in Appendix A.2, available as supplementary data at *Bioinformatics* online).

### 2.3 Hybrid models

Hybrid models combine sequence and structural information for enriched representations. DeepFRI (Gligorijević *et al.* 2021) combined LSTM-based sequence extraction with graph representations, while ESM-GearNet (Zhang *et al.* 2023c) explored different ways of combining ESM-1b with GearNet, setting new benchmarks across function prediction tasks. Recent approaches have focused on integrating geometric awareness into language models (Yuan *et al.* 2024, Tan *et al.* 2024, Wang *et al.* 2025). It is noteworthy that the follow-up to ESM-2, ESM-3 (Hayes *et al.* 2025) also integrates geometric and functional information into a transformer-based model for protein design, highlighting the importance of geometric information in PLMs. A comprehensive discussion of hybrid models is provided in Appendix A.3, available as supplementary data at *Bioinformatics* online. In contrast to previous approaches that merely attach structure-aware encoders to sequence-based models, our approach integrates structural information directly into *every* self-attention block of any transformer-based PLM. This strategy fosters a deeper integration of structural and sequential features, leading to models that outperform earlier methods in both accuracy and parameter efficiency. More details are provided in the next section to outline how this is achieved.

## 3 Methods

We first introduce our baseline backbone sequence models, ESM-2. Then we explain how to represent proteins as graphs and how to adapt the ESM-2 models to account for structural information.

### 3.1 Evolutionary scale modeling

ESM is a family of transformer PLMs, ranging from ESM-1b (Rives *et al.* 2021) (650M parameters) to the more advanced ESM-2 (Lin *et al.* 2023) (8M to 15B parameters). ESM-2 brings architectural enhancements and consistently outperforms ESM-1b on equal parameter grounds.

The ESM-2 language model employs masked language modeling, minimizing


(1) $$L_{\text{MLM}}=E_{x∼X}E_{M}\sum_{i\in M}-\text{log}\;p(x_{i}|x_{/M}),$$

where random amino acids in the sequence *x* are masked and the model predicts the original amino acid types from the unmasked context $x_{/M}$.

For the training phase, sequences are uniformly sampled across approximately 43 million UniRef50 training clusters, derived from around 138 million UniRef90 sequences. This ensures that during its training, the model is exposed to roughly 65 million distinct sequences.

#### 3.1.1 ESM-2 model architecture

The ESM-2 models use BERT-style encoder-only transformer architecture with certain modifications (Vaswani *et al.* 2017, Devlin *et al.* 2019). These models are constructed with multiple stacked layers, each comprising two primary building blocks: a self-attention layer followed by a feedforward layer. For the self-attention mechanism, token features $X\in R^{n\times d}$ are first projected to the query (*Q*), key (*K*), and value (*V*) matrices through linear transformations, as given by


(2) $$Q=XW_{Q},\;K=XW_{K},\;V=XW_{V},$$

where $W_{*}\in R^{d\times d_{\text{out}}}$ represent trainable parameters. The resulting self-attention is defined as


(3) $$\text{Attn}(X)=\text{softmax}\left(\frac{QK^{⊤}}{\sqrt{d_{\text{out}}}}\right)V\in R^{n\times d_{\text{out}}}.$$

It is worth noting that multi-head attention, which concatenates multiple instances of Equation (3), is commonly adopted and has been empirically effective (Vaswani *et al.* 2017). Then, the output of the self-attention is followed by a skip-connection and a feedforward network, which jointly compose a transformer layer, as shown below:


(4) $$\begin{array}{cc} X^{\prime } & =X+\text{Attn}(X),\\ X^{\prime \prime } & =X^{\prime }+\text{FFN}(X^{\prime }):=X^{\prime }+\text{ReLU}(X^{\prime }W_{1})W_{2}, \end{array}$$

where $W_{1}$ and $W_{2}$ are trainable parameters and ReLU denotes the rectifier activation function. While the original transformer uses absolute sinusoidal positional encoding to inform the model about token positions, ESM-2 leverages the Rotary Position Embedding (Su *et al.* 2024), enabling the model to extrapolate beyond its trained context window. The main goal of our PST is to slightly modify ESM-2 (or any other transformer-based PLM) such that it can take protein structural information into account.

### 3.2 Protein structure transformer

**Figure 1.**
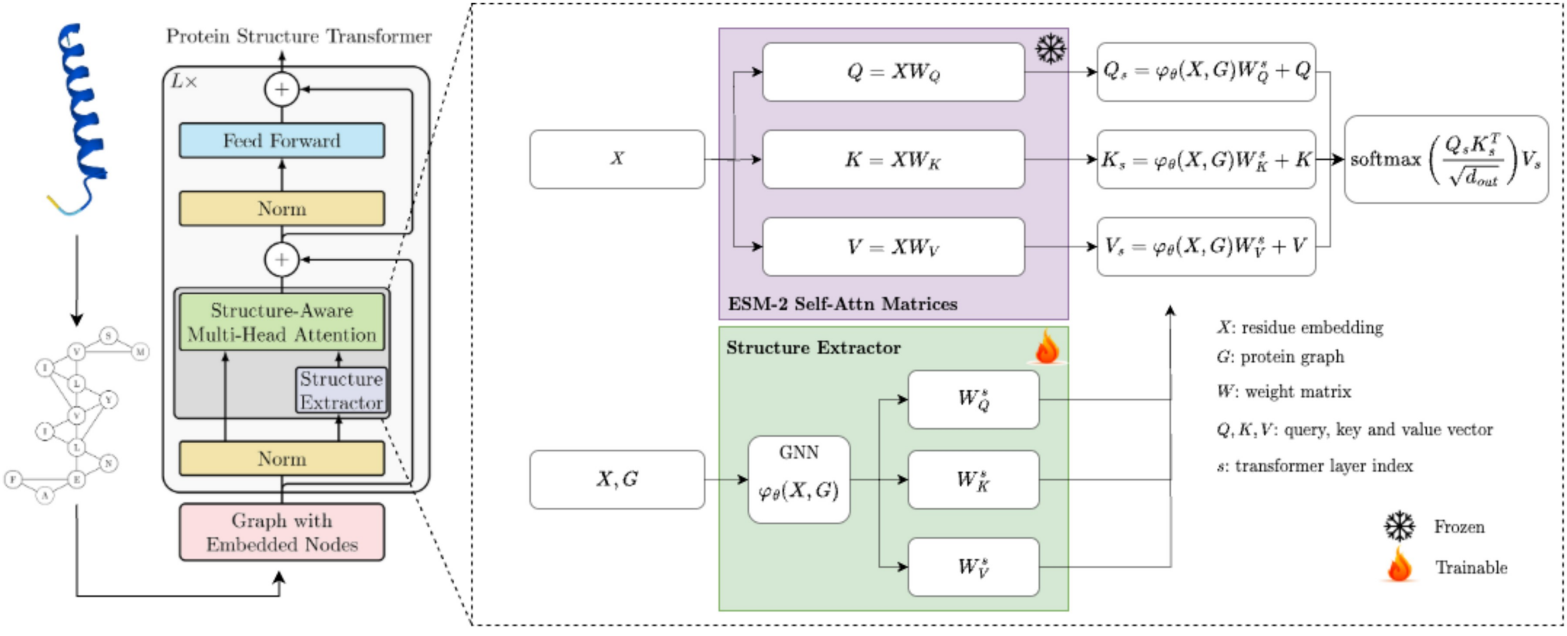
Overview of the proposed PST. A protein 3D structure is converted to an ordered graph with nodes representing amino acid types and edges linking any pair of residues within a specified threshold (8 Å). Then, we sample a fraction of nodes and mask them using a special mask token. The output of PST is fed into a linear prediction head to predict the original amino acid types. Compared to ESM-2, PST uses a GNN to extract local structural features around each node before computing the self-attention at each transformer layer. The PST model is initialized with the pretrained ESM-2 weights.

Recent advances in graph representation learning facilitated the adaptation of transformer architectures to process graph-structured data, leading to the emergence of what are formally termed “graph transformers.” In particular, structure-aware transformers (Chen *et al.* 2022) meld the vanilla transformer framework with GNNs. This integration proficiently captures complex interactions inherent to local structures, offering substantial improvement over conventional GNNs. Considering the intrinsic ability to represent protein structures as graphs, these advances position graph transformers as particularly adequate for modeling protein structures. In the following, we present the methodology for representing a protein as a graph and delineate the modifications implemented in the ESM-2 model to construct our dedicated PST. The model architecture and the complete pretraining process are illustrated in Fig. 1 and Fig. 1, available as supplementary data at *Bioinformatics* online, respectively. In summary, we employ a structure extractor, e.g., a shallow GNN, that modifies *each self-attention calculation* within the ESM-2 model. Note that the structure extractor may vary across layers. It takes the residue embedding from the specific layer it is applied to, along with structural information, and produces an updated residue embedding. This updated embedding is then transformed to update the query, key, and value matrices provided by the ESM-2 for the self-attention mechanism.

#### 3.2.1 Protein graph representation

Proteins can be conceptualized as ordered graphs, denoted as *G*. In this representation, the node order encodes the sequence annotated with amino acid types. A connection is drawn between two nodes when the spatial distance between the corresponding residues remains within a specified threshold. Based on our empirical studies, this threshold is set at 8.0 Å, primarily because a majority of local intermolecular interactions manifest within this range Bissantz *et al.* (2010). The residue distances might serve as potential edge attributes for the graph; however, omitting distance information proved more effective, as discussed in Section 4.4.

#### 3.2.2 Protein structure transformer construction

The PST, built upon an ESM-2 model, accepts the protein graph as input. A distinguishing feature is the substitution of traditional self-attention mechanisms with structure-aware attention mechanisms (Chen *et al.* 2022). Concretely, the residue embeddings at each self-attention layer, in conjunction with the graph structure encoding the protein structure, are processed through a structure extractor function $\varphi_{\theta }(X,G)\in R^{n\times d}$ to ascertain local structural contexts surrounding each residue, prior to computing the self-attention. These deduced structural embeddings subsequently undergo a linear transformation prior to being added to the query, key and value matrices, as expressed by


(5) $$\begin{array}{cc} Q_{s} & =Q+\varphi_{\theta }(X,G)W_{Q}^{s},\\ K_{s} & =K+\varphi_{\theta }(X,G)W_{K}^{s},\\ V_{s} & =V+\varphi_{\theta }(X,G)W_{V}^{s}. \end{array}$$

Here, $W_{*}^{s}\in R^{d\times d_{\text{out}}}$ signifies trainable parameters initialized at zero, ensuring that the resulting architecture, prior to any training, mirrors the base ESM-2 model. Pertinently, the structure extractor function $\varphi_{\theta }$ can be any function operating on the subgraphs around each node. For computational reasons, we select a commonly used graph neural network, specifically GIN (Xu *et al.* 2018) at *each self-attention block* of the ESM-2 model. The Each GIN has two layers, as suggested by Chen *et al.* (2022).

#### 3.2.3 Pretraining the PST

The PST, initialized with the pretrained weights of an ESM-2 model, is further pretrained on a protein structure database, employing the masked language modeling objective same as the base ESM-2 model. One can opt to either update solely the parameters within the structure extractors θ and $W_{s}$ or refine the entire model. Comparative evaluations of these strategies will be discussed in Section 4.4.

## 4 Experiments

We investigate PST’s applicability across downstream tasks with and without fine-tuning and analyze model architecture, pretraining strategy, and structural information requirements to identify performance drivers. We focus on the following aspects:

- First, we assess if PST can attain competitive performance on *function prediction* tasks.
- Next, we probe the robustness of its representations *without task-specific fine-tuning*.
- We then investigate if PST surpasses ESM-2, with emphasis on smaller-capacity models to gauge parameter efficiency and the contribution of structural information.
- In parallel, we analyze how injecting additional structural information during pretraining shapes downstream performance, considering potential constraints of masked language modeling.
- Finally, we test whether pretraining only the structure extractors can approach full-model pretraining while improving parameter efficiency.

### 4.1 Experimental setup

#### 4.1.1 Function prediction datasets

We evaluate PST models using several benchmark datasets covering diverse tasks and compare against multiple baselines. We predict protein function from experimentally resolved structures using Gene Ontology (GO) terms or Enzyme Commission (EC) numbers. Dataset curation follows (Gligorijević *et al.* 2021). We calculate *F*${\;}_{\max}$ and the area under the precision recall curve (AUPR) scores and include fold classification from Hou *et al.* (2018). More information on those datasets and metrics is provided in Appendix C.5 and C.2, available as supplementary data at *Bioinformatics* online.

#### 4.1.2 ProteinShake benchmark datasets

ProteinShake (Kucera *et al.* 2023) provides evaluation across diverse biological tasks including EC, GO, protein family (Pfam) (Mistry *et al.* 2021), SCOPe (Chandonia *et al.* 2022), and binding site classification (Gallo Cassarino *et al.* 2014). These tasks encompass functional and structural classification at the protein-level, as well as residue-level classification for identifying binding pocket residues (Gallo Cassarino *et al.* 2014). The library provides metrics and precomputed splits based on sequence and structural similarity for each task. We exclusively use the most stringent structure-based splits.

#### 4.1.3 Variant effect prediction datasets

A common use of protein representations is to predict mutation effects, saving valuable bench time (Riesselman *et al.* 2018, Meier *et al.* 2021, Notin *et al.* 2023). We use computationally predicted AlphaFold structures as input, swapping each mutated position in the resulting graph before computing the protein’s representation. We evaluate on 38 proteins from Riesselman *et al.* (2018) and Meier *et al.* (2021), using Spearman’s correlation coefficient ρ between predicted scores and experimental outcomes as our benchmark metric. All predictions are made *without fine-tuning*, enabling fair comparison of PST and ESM-2 representation quality. Additional results are in Appendix C.6, available as supplementary data at *Bioinformatics* online.

#### 4.1.4 Pretraining

We build PST models on ESM-2 variants (esm2_t6_8M_UR50D, esm2_t12_35M_UR50D, esm2_t30_150M_UR50D, esm2_t33_650M_UR50D). Larger models exceeded GPU VRAM. For each ESM-2 model, we endow it with structural knowledge by incorporating the structure extractor in every self-attention block, as described in Section 3.2.2. We use GIN (Xu *et al.* 2018) as our structure extractor with two GNN layers, as suggested in Chen *et al.* (2022). We pretrain all four PST models on AlphaFold’s SwissProt subset of 542 378 predicted structures (Jumper *et al.* 2021, Varadi *et al.* 2022). We initialize the model weights with the corresponding pretrained ESM-2 weights, except for the structure extractors that are initialized randomly (for θ) or to zeros for the linear projection parameters $W_{*}^{s}$.

#### 4.1.5 Task-specific models

To assess representation generalizability efficiently, we fix the representations across tasks instead of performing task-specific fine-tuning. We average the residue-level representations after each transformer block, and concatenate them to obtain protein-level embeddings. We choose an MLP for multi-label classification and a linear model for other types of classification.

#### 4.1.6 Training details and hyperparameter choice

For the pretraining of PST models, we use AdamW with linear warmup and inverse square root decay (Vaswani *et al.* 2017, Rives *et al.* 2021). Training time is 10 hours per model on 4 H100 GPUs. Other hyperparameters for optimization are provided in Appendix C.4, available as supplementary data at *Bioinformatics* online.

After pretraining, we can extract and fix representations of proteins. Once the representations are extracted, we train a task-specific classifier. These classifiers are chosen to be an MLP for multi-label classification and a linear model for other types of classification. The MLPs consist of three layers, with the dimension of each hidden layer reduced by a factor of 2 each time. We also use a dropout layer after each activation, with its dropout ratio to be 0 or 0.5, optimized on the validation set. Each model is trained for 100 epochs using the binary cross-entropy loss, the AdamW optimizer, and the learning rate is reduced by a factor of 0.5 if the validation metric of interest is not increasing after 5 epochs. All hyperparameters are selected based on the validation set.

### 4.2 PST without fine-tuning adapts to diverse tasks

In this section, we evaluate the performance of PST models against several state-of-the-art counterparts on several function and structure prediction datasets, as shown in Table 1. The comparison partners include sequence models without pretraining such as CNN (Shanehsazzadeh *et al.* 2020), Transformer (Rao *et al.* 2019), with pretraining such as ProtBERT-BFD (Elnaggar *et al.* 2021), Ankh (Elnaggar *et al.* 2023), ESM-1b (Rives *et al.* 2021), ESM-2 (Lin *et al.* 2023), structure models with pretraining such as DeepFRI (Gligorijević *et al.* 2021), LM-GVP (Wang *et al.* 2022), GearNet multiview contrast (MVC) (Zhang *et al.* 2023a), and a hybrid model ESM-GearNet MVC (Zhang *et al.* 2023c), which integrates ESM-1b and GearNet MVC, as well as its newer versions ESM-2-Gearnet MVC and ESM-2-Gearnet SiamDiff, introduced in Zhang *et al.* (2023b). Section E.6, available as supplementary data at *Bioinformatics* online adds a comparison to an alignment-based baseline.

**Table 1.** Performance of PST models compared to other sequence or structure-aware embedding methods on protein function prediction tasks. Bold values indicate the best performer for a metric and task combination.^a^

Method	EC		GO-BP		GO-MF		GO-CC		Fold class (ACC)		
	*F* ${\;}_{\max}$	AUPR	*F* ${\;}_{\max}$	AUPR	*F* ${\;}_{\max}$	AUPR	*F* ${\;}_{\max}$	AUPR	Fold	Super.	Fam.
End-to-end training											
DeepFRI	0.631	0.547	0.399	0.282	0.465	0.462	0.460	0.363	15.3	20.6	73.2
ESM-1b	0.864	0.889	0.452	0.332	0.657	0.639	0.477	0.324	26.8	60.1	97.8
ProtBERT-BFD	0.838	0.859	0.279	0.188	0.456	0.464	0.408	0.234	26.6	55.8	97.6
LM-GVP	0.664	0.710	0.417	0.302	0.545	0.580	**0.527**	**0.423**	–	–	–
GearNet MVC	0.874	0.892	0.490	0.292	0.654	0.596	0.488	0.336	**54.1**	80.5	**99.9**
ESM-1b-Gearnet MVC	0.894	0.907	**0.516**	0.301	**0.684**	0.621	0.506	0.359	–	–	–
ESM-2-GearNet MVC	0.896	–	0.514	–	0.683	–	0.497	–	–	–	–
ESM-2 (fine-tuned)	0.861	0.871	0.460	0.308	0.663	0.627	0.427	0.331	38.5	81.5	99.2
PST (fine-tuned)	**0.897**	**0.919**	0.489	**0.348**	0.675	**0.648**	0.475	0.375	42.3	**85.9**	99.7
Fixed representations + classification head											
Ankh	0.870	0.897	0.466	0.347	0.650	**0.647**	0.500	0.393	33.3	74.9	98.7
GearNet MVC	0.826	0.852	0.428	0.303	0.594	0.589	0.433	0.337	38.5	68.3	98.8
ESM-2	0.892	0.910	0.509	0.355	**0.686**	0.629	0.529	**0.394**	39.7	80.4	98.8
PST	**0.899**	**0.918**	**0.513**	**0.371**	**0.686**	0.637	**0.541**	0.387	**40.9**	**83.6**	**99.4**

a Relevant comparison partners are selected from Zhang *et al.* (2023c) and Elnaggar *et al.* (2023).

Unlike previous studies focusing on training independent models for each individual task, we take a distinct approach, aiming to assess the universality of protein representations from pretrained models. For this, we fix the representations across tasks following the procedure described in Section 4.1.4. This procedure is equally applied to both GearNet MVC and ESM-2. Contrary to the claims in the work by Zhang *et al.* (2023a), our analysis suggests that ESM outperforms GearNet MVC in the sense that it generates more general-purpose representations. Notably, our PST models surpass ESM-2 in performance, particularly evident in the fold classification task, where PST models outperform ESM-2 significantly, underscoring the effectiveness of incorporating structural information in discerning protein structural variations.

We next investigate task-specific fine-tuning of our PST models. Though computationally more intensive than employing fixed representations, the fine-tuned PST models surprisingly do not achieve superior performance. Furthermore, PST models with fixed representations demonstrate competitive or superior performance against almost all end-to-end models while demanding substantially reduced computational time, emphasizing their enhanced adaptability and applicability in diverse real-world scenarios. It is worth noting that PST surpasses ESM-GearNet MVC, a model that integrates ESM in a decoupled fashion.

### 4.3 PST consistently outperforms ESM-2

In this section, we provide further evidence that our PST model consistently outperforms the ESM-2 model on which it is based. As highlighted in Section 4.2, across a wide range of function prediction tasks, employing PST embeddings alongside an MLP classification head consistently achieves enhanced performance, relative to using ESM embeddings with a comparable MLP head.

To mitigate the potential influences of optimization and inherent randomness in training the MLP, we opt for a simple linear model over the representations for a variety of tasks from ProteinShake to better assess representation quality. As seen in Table 2, PST exhibits consistent superiority over ESM-2 across these tasks. This distinction is particularly pronounced in the EC classification, where PST markedly surpasses ESM-2.

**Table 2.** Comparison of PST and ESM-2 on ProteinShake tasks and VEP datasets.^a^

Method	GO	EC	Protein family	Binding site	Structural class	Zero-shot VEP
	*F* ${\;}_{\max}$	ACC	ACC	MCC	ACC	Mean |ρ|
ESM-2	0.648	0.858	0.698	0.431	0.791	0.489
PST	0.650	0.883	0.704	0.436	0.797	0.501

a Details of evaluation metrics can be found in Appendix C.2, available as supplementary data at *Bioinformatics* online.

Additionally, we compare PST with ESM-2 in the context of zero-shot variant prediction tasks, conducted *without any further tuning*. PST equally outperforms ESM-2 in terms of average Spearman’s rank correlations. The better results in tasks like binding site detection and variant effect prediction imply that PST not only offers enhanced protein-level representations but also refines residue-level representations.

### 4.4 Ablation studies

While Section 4.3 showcases the added value of PST relative to its base ESM-2 model, we now dissect the components of PST to identify what drives the performance.

#### 4.4.1 Simpler structural inputs yield better performance

We start the analysis by investigating the extent of structural information required for refining the ESM-2 models. For this purpose, we construct two ϵ-neighborhood graphs from protein 3D structures: one that does not incorporate distance information as edge attributes, and another enriched with 16-dimensional features related to residue distances serving as edge features. Structure extractors are then adapted accordingly to account for edge attributes. Details about these features can be found in the Appendix C.7, available as supplementary data at *Bioinformatics* online. Subsequently, PST models, equipped with structure extractors either with or without edge attributes are pretrained. Their generated representations are then assessed using the ProteinShake datasets.

**Figure 2.**
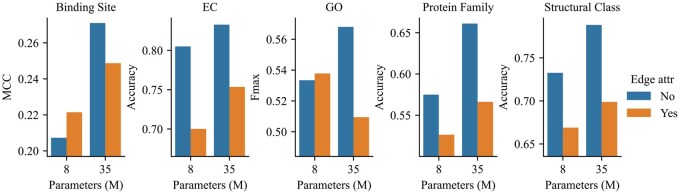
Performance of PST models trained with and without distance information used as edge attributes on ProteinShake tasks.

While incorporating more granular structural information augments pretraining accuracy (e.g., from 47% to 55% for the six-layer models), it does not translate to better downstream performance (potentially due to overfitting), as depicted in Fig. 2. We note that the PST model leveraging edge attributes underperforms its counterpart without edge attributes across all the tasks. Such a phenomenon could plausibly stem from the inherent simplicity of the masked language modeling objective, suggesting a potential necessity to devise more nuanced objectives when integrating advanced structural features.

#### 4.4.2 PST shows largest gains in smaller models

We evaluate PST models across various model sizes and measure the performance uplift relative to their base ESM-2 model. We pretrained four distinct PST models, each based on the ESM-2 models with sizes spanning 8M, 35M, 150M, and 650M parameters. Owing to our employment of shallow, lightweight GINs as structure extractors, the resulting PST models maintain a parameter count that is less than double that of their base ESM-2 models.

**Figure 3.**
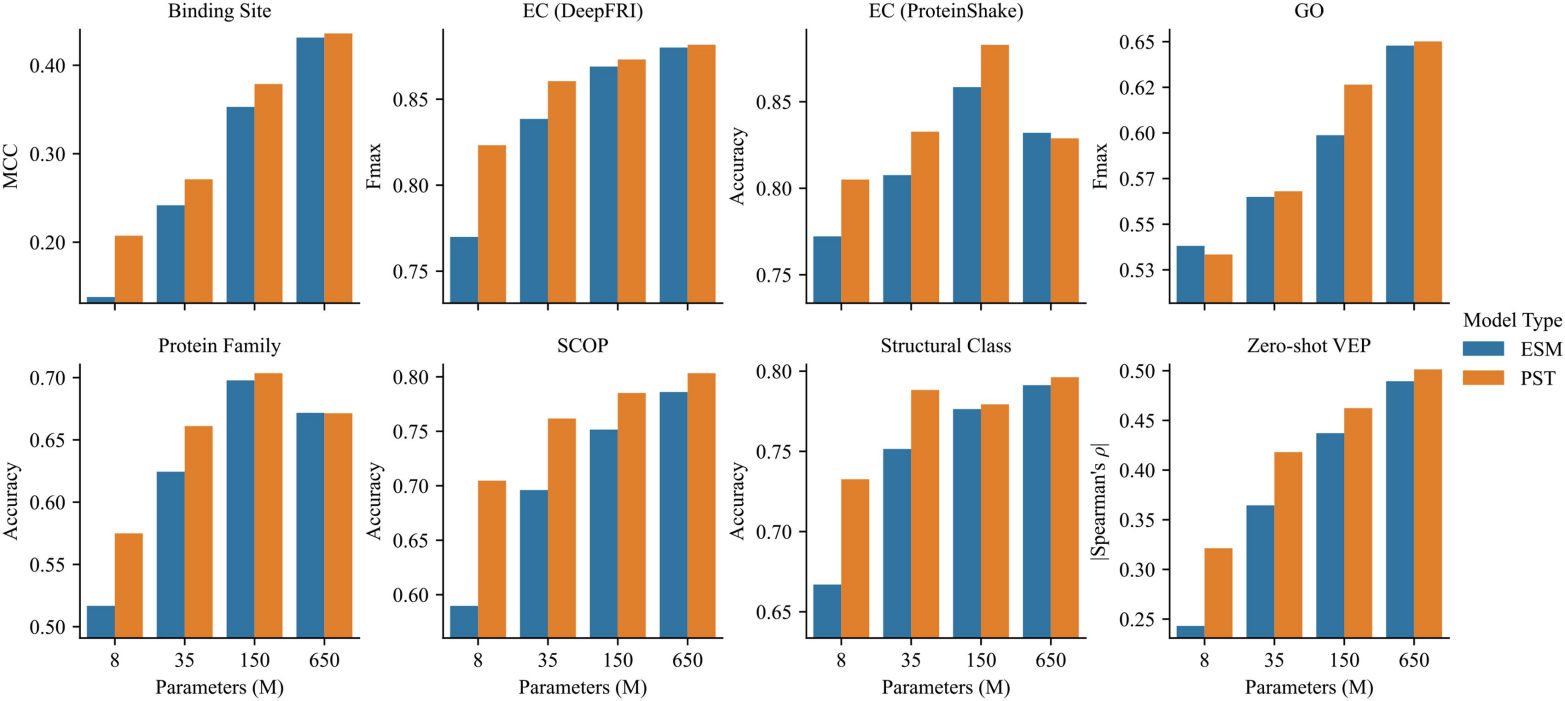
Performance of PST and ESM-2 across varied model sizes on ProteinShake datasets as well as DeepFRI and variant effect prediction (VEP) datasets.

Figure 3 presents the outcomes of our assessment. Notably, as model size increases, both ESM-2 and PST display enhanced performance across the majority of tasks, with exceptions observed in EC (ProteinShake) and Protein Family classification. While PST typically surpasses its base ESM-2 counterpart at similar model sizes, this performance gain tapers off with increasing model size. This trend is potentially due to the increased capacity of larger ESM models, allowing them to infer protein structures from sequences alone (Lin *et al.* 2023). Consequently, for scenarios constrained by computational resources, opting for structure-aware models might offer a strategic advantage.

#### 4.4.3 Pretraining only structure extractors almost matches full-model pretraining performance

**Figure 4.**
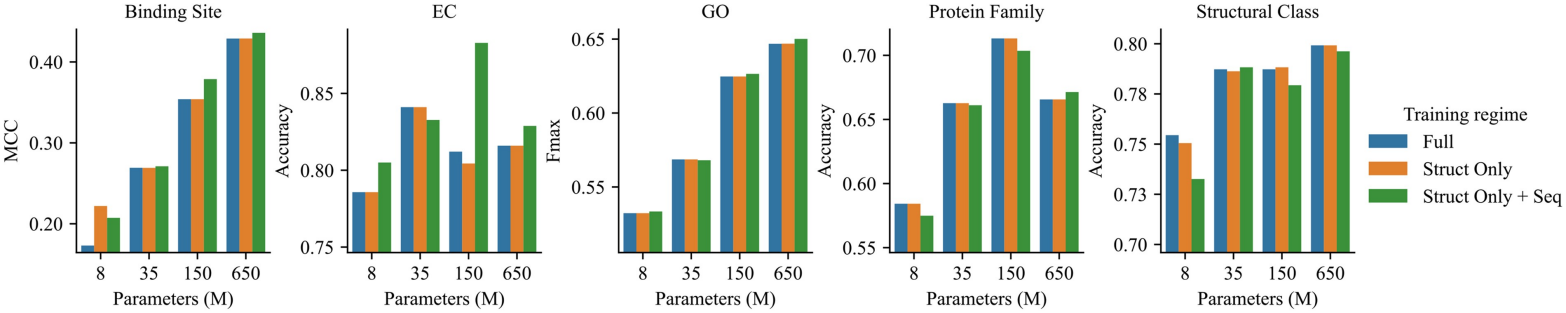
Effect of pretraining strategies on model performance. “Full” refers to the strategy where one updates the full model during pretraining, including both ESM-2 and structure extractor weights. “Struct Only” refers to the strategy where only the structure extractor weights are being updated during training. “Struct Only + Seq” is an extension of “Struct Only” *at inference*. By bypassing the structure extractors, the PST model is capable of obtaining the same sequence representations as the base ESM-2 model. Averaging both structure and sequence representations leads to “Struct Only + Seq.”

In Section 3.2.2, we delineate the distinctions between PST and ESM-2 models, pinpointing the addition of structure extractors as the only difference. Here, our experiments seek to ascertain if solely updating the structure extractors yields comparable results to a full-model pretraining. Figure 4 shows a performance comparison on ProteinShake tasks, where the orange bars (“Struct Only”) correspond to the pretraining strategy only updating the structure extractors, and the blue bars (“Full”) represent full-model updates during pretraining.

Remarkably, pretraining restricted to the structure extractors produces performance outcomes akin to a full-model pretraining. Beyond parameter efficiency, this selective updating confers an additional advantage: the capability to derive the base ESM-2 model’s sequence representation from the same model at inference, achieved through bypassing the structure extractors. By averaging both structure and sequence representations, we obtain enhanced representations beneficial for multiple tasks, as depicted by the “Struct Only + Seq” (green bars) in Fig. 4.

#### 4.4.4 PST gains are due to structural information

To verify that PST’s performance gains stem from structural information rather than additional parameters, we perturbed the graph in three ways: removing non-sequential edges, random rewiring, and using a fully connected graph. These perturbations effectively eliminate secondary/tertiary structure while maintaining parameter count. Results in Fig. 4 (Appendix E), available as supplementary data at *Bioinformatics* online confirm that structural information, not additional parameters, drives PST’s improvements.

#### 4.4.5 PST effectively uses predicted structures

We evaluated PST’s robustness to structure quality by testing it on ProteinShake benchmarks using both experimental (PDB) and predicted (AlphaFold 2, ESM-2) structures. Results in Fig. 5 (Appendix E), available as supplementary data at *Bioinformatics* online show PST maintains performance across different structure sources.

#### 4.4.6 Additional ablations

Sections E.3 to E.5, available as supplementary data at *Bioinformatics* online show that downstream performance is largely unaffected by the number of GIN layers, the threshold for extracting graphs from protein coordinates, or whether edges are defined by spatial proximity or sequence.

## 5 Discussion

In this work, we presented PST, a novel, easy-to-use and lightweight framework that endows pretrained PLMs with structural knowledge. Unlike previous models requiring training from scratch, our approach refines existing transformer-based PLMs, amplifying their accumulated sequence knowledge.

Our evaluations reveal that PST generates general-purpose protein representations, excelling across function prediction tasks and surpassing the cutting-edge sequence-based model ESM-2 in various protein property prediction tasks.

Our analysis reveals that large sequence-based PLMs can recover implicit structural information from sequences alone. However, only large models exhibit this ability. PST compensates for smaller models by providing minimal structural input, enabling structure awareness and boosting performance.

While powerful and lightweight, our work is currently constrained to pretraining on 542K protein structures, while the AlphaFold Protein Structure Database contains over 200 million structures, presenting significant opportunities for advanced structure-based models. We envision that using more structural data and advanced pretraining objectives beyond traditional masked language modeling will unlock the full potential of larger models within the PST paradigm.

## Supplementary Material

btaf582_Supplementary_Data

## Data Availability

All the data, code and model checkpoints used for this publication is available at https://github.com/BorgwardtLab/PST
