# Supplementary material for: Endowing protein language models with structural knowledge
Source: Bioinformatics. 2025 Oct 24;41(11):btaf582. doi: 10.1093/bioinformatics/btaf582 (PMC12603367; doi:10.1093/bioinformatics/btaf582)
Supplement: btaf582_Supplementary_Data [file btaf582_supplementary_data.pdf]

Appendix to "Endowing Protein Language Models with Structural Knowledge"

Philip Hartout[1], Dexiong Chen[1], Paolo Pellizzoni[1], Carlos Oliver[1], Karsten Borgwardt[1]

[1]: Department of Machine Learning and Systems Biology, Max Planck Institute of Biochemistry, Am Klopferspitz 18, 82152 Martinsried, Bavaria, Germany

Additional Related Work

Additional Sequence-based Models

Beyond the transformer-based models highlighted in the main text, several other sequence-based approaches have made notable contributions to protein representation learning. Shanehsazzadeh *et al.* (2020) demonstrated that even without pretraining, CNNs can surpass the performance of pretrained transformers on certain protein tasks. Additional protein language models include xTrimoPGLM (Chen *et al.*, 2023) and Ankh (Elnaggar *et al.*, 2023), which have further enhanced modeling capabilities within the protein domain. Other notable work in sequence-based protein modeling includes (Lin *et al.*, 2023; Hu *et al.*, 2022). ESM Cambrian (ESM Team, 2024) and ProGen have also been proposed as a recent PLM (Madani *et al.*, 2023; Nijkamp *et al.*, 2023; Bhatnagar *et al.*, 2025), but they are much larger models than the smaller ESM-2 family of models.

Additional Structure-based Models

Structure-based protein modeling has employed diverse architectural approaches beyond the GNN-based methods mentioned in the main text. Early approaches included 3D Convolutional Neural Networks (CNNs) (Derevyanko *et al.*, 2018), as well as point cloud and voxel-based models (Yan *et al.*, 2022; Mohamadi *et al.*, 2022). The theoretical foundations of graph-based representations for proteins have been explored by Xu *et al.* (2018).

Additional GNN-based approaches include (Hermosilla *et al.*, 2021), which offers inherent flexibility to encode protein-specific features. Methods employing contrastive learning for 3D protein structures have been developed by Hermosilla and Ropinski (2022). Graph attention networks have been utilized for protein function prediction, as demonstrated by Lai and Xu (2022). Powerful language models based on a tokenized vocabulary have also shown promising results such as SaProt (Su *et al.*, 2024) and further explored and benchmarked in Gaujac *et al.* (2024).

Additional Hybrid Models

The integration of sequence and structural information has been explored through various hybrid architectures beyond those highlighted in the main text. Wang *et al.* (2022) utilized ProtBERT-BFD embeddings as input features for the GVP model, resulting in enhanced predictive capabilities. Zheng *et al.* (2023) explored structure-informed language models specifically for protein design tasks, with a focus on inverse protein folding. A systematic study on joint representation learning from protein sequences and structures was conducted by Zhang *et al.* (2023).

Recent hybrid approaches have included MEGA-GO by Lee *et al.* (2025), a multi-scale graph adaptive neural network for predicting functions of proteins with varying sequence lengths. PANDA2 (Zhao *et al.*, 2022) and Struct2GO (Jiao *et al.*, 2023), which utilizes graph pooling and AlphaFold2 structural data, have also contributed to this space. GGN-GO (Mi *et al.*, 2024) features geometric graph networks with multi-scale structural features, while PANDA-3D (Zhao *et al.*, 2024) focuses on function prediction from AlphaFold models.

Pipeline overview

An overview of the pipeline described in this work is provided in Figure S1.

Experimental Details and Additional Results

Computation Details

We perform all the pretraining of PST models on 4 H100 GPUs. 20GiB H100 MIGs were used to fine-tune the classification heads.

Evaluation metrics

In this Section, we briefly describe the performance metrics we used in the experimental evaluation of the models.

Protein-centric F_{max} score

The $\mathrm{F_{max}}$ score is computed as follows. Let $P_x(\tau)$ be the positive functions for the protein x, i.e. the

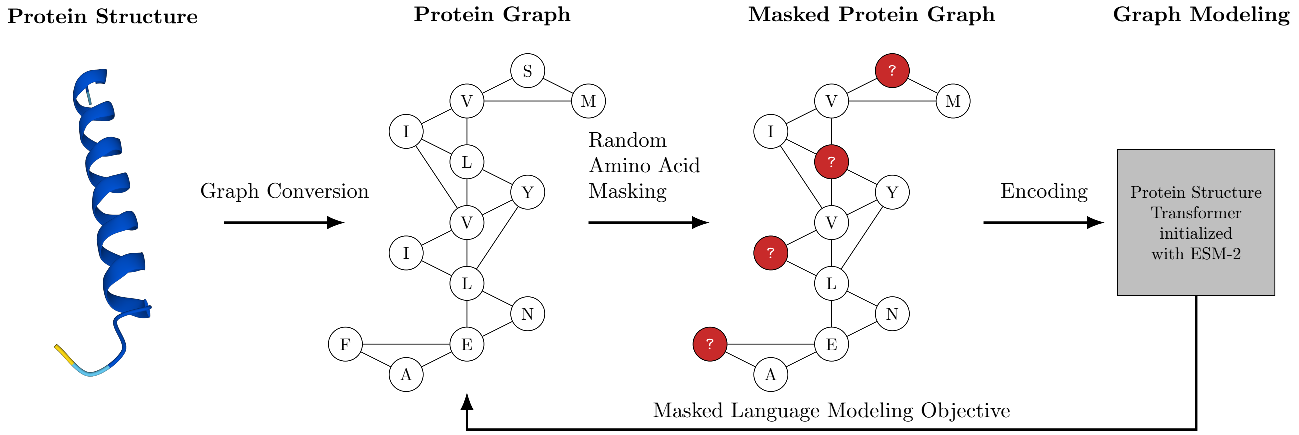

Fig. S1. Overview of the pretraining pipeline of the proposed Protein Structure Transformer.

Precision (pr_i) and recall (rc_i) for a given protein i at threshold τ are defined as

$$pr_i(\tau) = \frac{\sum_f \mathbb{1}(f \in P_i(\tau) \cap T_i)}{\sum_f \mathbb{1}(f \in P_i(\tau))},$$

$$rc_i(\tau) = \frac{\sum_f \mathbb{1}(f \in P_i(\tau) \cap T_i)}{\sum_f \mathbb{1}(f \in T_i)},$$

where $P_i(\tau)$ is the set of terms that have predicted scores greater than or equal to τ for a protein i and T_i denotes the ground-truth set of terms for that protein. Then the set $P_i(\tau) \cap T_i$ is the set of true positive terms for protein i.

The average precision and average recall are then defined as

$$pr(\tau) = \frac{1}{n(\tau)} \sum_{i=1}^{n(\tau)} pr_i(\tau),$$

$$rc(\tau) = \frac{1}{N} \sum_{i=1}^{N} rc_i(\tau),$$

where N is the number of proteins and $n(\tau)$ is the number of proteins with at least one predicted score greater than or equal to τ.

Then, we define the $\mathrm{F_{max}}$ score as

$$\mathrm{F_{max}} = \max_{\tau} \left\{ \frac{2 \cdot pr(\tau) \cdot rc(\tau)}{pr(\tau) + rc(\tau)} \right\}.$$

Pair-centric AUPR

The AUPR score we use in the evaluation, following Zhang *et al.* (2022) is computed by area under the precision-recall curve, is defined as the average, over all protein-term pairs (i, f), of the AUPR score of the classifier on that binary classification task.

MCC

Matthews correlation coefficient, abbreviated MCC, is defined, for a binary classification task, as follows. Let TP, FP, TN and FN be the number of true positives, false positives, true negatives and false negatives, respectively. Then we define

$$\mathrm{MCC} = \frac{TP \cdot TN - FP \cdot FN}{\sqrt{(TP + FP)(TP + TN)(TN + FN)(FP + FN)}}.$$

A score of 1 indicates perfect agreement between prediction and labels, a score of 0 indicates no correlation among the two, and a score of -1 indicates perfect disagreement.

Spearman's rank correlation coefficients ρ

Spearman's rank correlation coefficient can be calculated using:

$$\rho_S = 1 - \frac{6 \sum d_i^2}{n(n^2 - 1)}$$

where $d_i = \mathrm{R}(X) - \mathrm{R}(Y)$ is the difference between two ranks of each observation. $\rho_S \approx 1$ or $\rho_S \approx -1$ indicates that the ranks of the two observations have a strong statistical dependence, $\rho_S \approx 0$ indicates no statistical dependence between the ranks of two observations. In this work, we look at $|\rho_S|$, as 1 and -1 carry the same meaning in the context of zero-shot VEP. See Appendix C.6 for more details.

Details of Baselines

We took performance values from Zhang *et al.* (2022) for the following baselines: CNN, Transformer, DeepFRI, ESM-1b, ProtBERT-BFD, LM-GVP, GearNet MVC (end-to-end training), ESM-Gearnet MVC. Moreover, we fine-tuned ESM-2 ourselves on the tasks at hand. Finally, we used the embeddings obtained from the Ankh, GearNet MVC and ESM-2 models as input to a MLP classification head.

Table S1 reports the number of parameters for the models that we trained ourselves. We were not able to find the number of parameters for the other baselines.

Table S1. Number of parameters of the baseline models.

Model name	Number of parameters
GearNet MVC	20 M
Ankh	1151 M
ESM-2 (`esm2_t33_650M_UR50D`)	651 M
PST (`esm2_t33_650M_UR50D`)	1137 M (where 486 M were trainable parameters)

Additional PST Hyperparameters

Table S2 reports additional hyperparameter choices we used for the pretraining of PST models.

Table S2. Hyperparameters used for pretraining PST models.

Model name	Learning rate	Batch size	Epochs	Warmup epochs
`esm2_t6_8M_UR50D`	0.0003	128	50	5
`esm2_t12_35M_UR50D`	0.0001	64	20	5
`esm2_t30_150M_UR50D`	5e-5	16	10	1
`esm2_t33_650M_UR50D`	3e-5	12	5	1

Details of Function Prediction and Structural Classification Datasets

Function prediction

Similar to (Gligorijević *et al.*, 2021) and (Zhang *et al.*, 2022), the EC numbers are selected from the third and fourth levels of the EC tree, since the EC classification numbers are hierarchical. This forms a set of 538 binary classification tasks, which we treated here as a multi-label classification task. GO terms with training samples ranging from 50 to 5000 training samples are selected. The sequence identity threshold is also set to 95% between the training and test set, which is again in line with Gligorijević *et al.* (2021); Zhang *et al.* (2022) and Wang *et al.* (2022). Note that Zhang *et al.* (2022) also performs additional quality control steps in their code, and to ensure a fair comparison we extracted the graphs and sequences directly from their data loader.

Fold classification

Like (Zhang *et al.*, 2022), we follow (Hou *et al.*, 2018) to predict the SCOPe label of a protein. There are three different splits that we use for predicting the label: *Fold*, where proteins from the same superfamily are unseen during training; *Superfamily*, in which proteins from the same family are not present during training; and *Family*, where proteins from the same family are present during training.

Table S3. Dataset statistics for downstream tasks.

**Dataset**	**Train**	**Validation**	**Test**
			Fold / *Superfamily* / *Family*
**Enzyme Commission**	15,550	1,729	1,919
**Gene Ontology**	29,898	3,322	3,415
**Fold Classification**	12,312	736	718 / 1,254 / 1,272

Variant Effect Prediction Datasets

A set of deep mutational scans (DMS) from diverse proteins curated by Riesselman *et al.* (2018) are used to benchmark sequence-based models and PST, since it was used to evaluate state-of-the-art models (Meier *et al.*, 2021). DMS experiments are routinely used in biology to assess the sequence-function landscape of a protein by mutating one or more of its residues and evaluating the resulting mutant function with respect to the wild type protein. The function of a protein can be measured using various

mechanisms, such as growth of the cell in which the (mutated) protein is expressed, resistance to antibiotics, or fluorescence, depending on the protein and experimental conditions (Fowler and Fields, 2014). In this collection of DMS experiments, a wide variety of readouts and proteins are evaluated depending on the task, and they are summarized in Supplementary Table 1 in Riesselman *et al.* (2018). Let G be the graph representation of the wild type protein structure, x^{mut} and x^{wt} be the mutant and wild type sequences, respectively. Let $x_{/M}$ be a sequence with masked node indices M, and M the total set of mutations applied to a single wild type protein. Then, the log masked marginal probability is defined as:

$$\sum_{i\in M} \log p(x_i = x_i^{\mathrm{mut}} | x_{/M}, G) - \log p(x_i = x_i^{\mathrm{wt}} | x_{/M}, G). \qquad (6)$$

This procedure allows us to perform mutational effect prediction without any additional supervision or experimental data on each task, deriving knowledge solely from the semi-supervised pretraining process. * We then compute the Spearman correlation coefficient between this set of scalar values obtained for each mutation and the observed effect in the variable of interest (depending on the protein).

Figure S2 shows the performance of ESM-2 and PST representations on deep mutational scan datasets collected by Riesselman *et al.* (2018). The standard deviations are obtained using 20 bootstrapped samples, in accordance with previous work (Meier *et al.*, 2021). The mean score between the ESM-2 score and PST were used for obtaining the PST model.

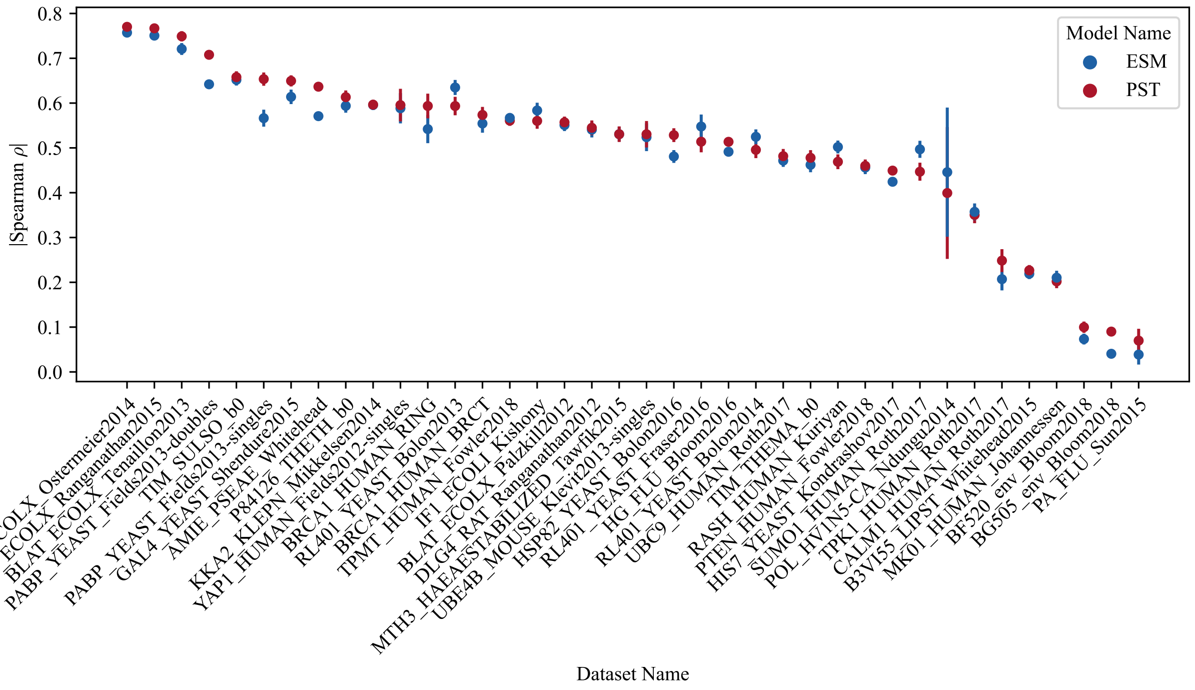

Fig. S2. 33-layer ESM and PST model performance on deep mutational scanning tasks.

Edge attributes details

We follow (Ingraham *et al.*, 2019) and extract 16 Gaussian RBFs isotropically spaced from 0 to 20 Å. This maximizes the value of the RBF kernel when the interatomic distance is close to the value of the center in the RBF kernel.

Illustration of Different Pretraining Strategies

The different pretraining strategies studied in section 4.4 are illustrated in Figure S3.

Additional Results

Corrupting structural information

Here we present the results of perturbing the underlying structure graph as an input to PST during pretraining and inference to investigate if the additional GNN parameters of PST are responsible for the performance increase, or if those are due to the

*We substitute the amino acid label in the wild-type structure, as AlphaFold2 authors explicitly said that point mutant structures are unreliable. See (Jumper *et al.*, 2021) for more details.

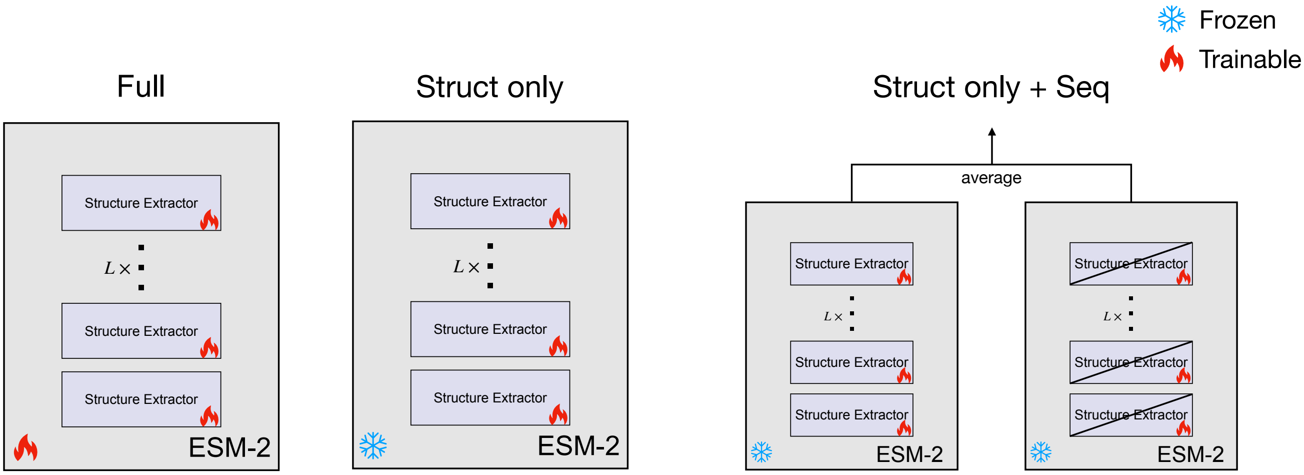

Fig. S3. Illustration of different pretraining strategies: "Full" refers to the strategy where one updates the full PST model including both ESM-2 and structure extractor weights. "Struct Only" refers to the strategy where only the structure extractor weights are being updated during training. "Struct Only" employs the same pretraining strategy as "Struct Only", and the only difference lies in the inference. Specifically, by bypassing the structure extractors, the PST model trained with the "Struct Only" strategy is capable to obtain the base ESM-2's sequence representations. By averaging both structure and sequence representations, we obtain the "Struct Only + Seq" strategy.

underlying structural information those parameters enable the model to extract. Those results are shown in Figure S4. Those results indicate that the gains in performance in PST are indeed due to the underlying structural information extracted by the GNNs and not due to the additional parameters, as evidenced by the consistently superior baseline performance over any of the corrupted models.

Swapping structure for ESM or AF2-generated structures

In practice, one might not always have access to the full structure. As a result, we aim to investigate the effect of inputting alternative structure sources to our model to investigate how well it performs. We see that our model is quite robust to such changes, the results are shown in Figure S5.

Differentiating between spatial edges and sequence edges very marginally impacts performance

We investigated if considering additional structural inductive biases, such as adding edge attributes specifying whether a given edge is an edge between two amino acids or resulting from spatial proximity. In figure S6, we show the results of those experiments. We show that, remarkably, adding those features does not confer additional discriminatory power, except for the enzyme classification task, where there is some moderate effect.

Increasing the number of GIN layers does not bring substantial performance improvements

Varying the number of GIN layers in PST does not seem to bring substantial performance improvements compared to the additional associated computational burden as can be seen in figure S7. This experiment was conducted with the 6-layer ESM-2 model as a backbone.

8.0 Å is appropriate as the threshold used for building graphs

We motivated our choice for the spatial distance threshold in Section 3.2.1 of 8.0 Å. Yet we investigated if varying this threshold would bring performance improvements. The results are shown in Figure S8. Those results show that the performance of PST is not very sensitive to the threshold used for graph construction, and in fact show that 8.0 Å is a good choice for constructing protein graphs, balancing graph density and computational efficiency with expressivity.

Comparison to an alignment-based baseline

Here, we compare our model to a non–deep-learning baseline that pairs MMseqs2 (Steinegger and Söding, 2017) sequence search with KNN: we search validation/test queries against the training set, keep the best hit per target, form a sparse similarity matrix, and predict labels with a KNN algorithm, selecting K based on cross-validation. This is a strong homology-transfer baseline; deep models should surpass it by capturing signals beyond nearest-neighbor sequence similarity, especially under low identity/remote homology. Tables S4 and S5 show these results. Note that the F_{max} can look high because it chooses the best threshold post hoc, whereas AUPRC averages over all thresholds and recall levels.

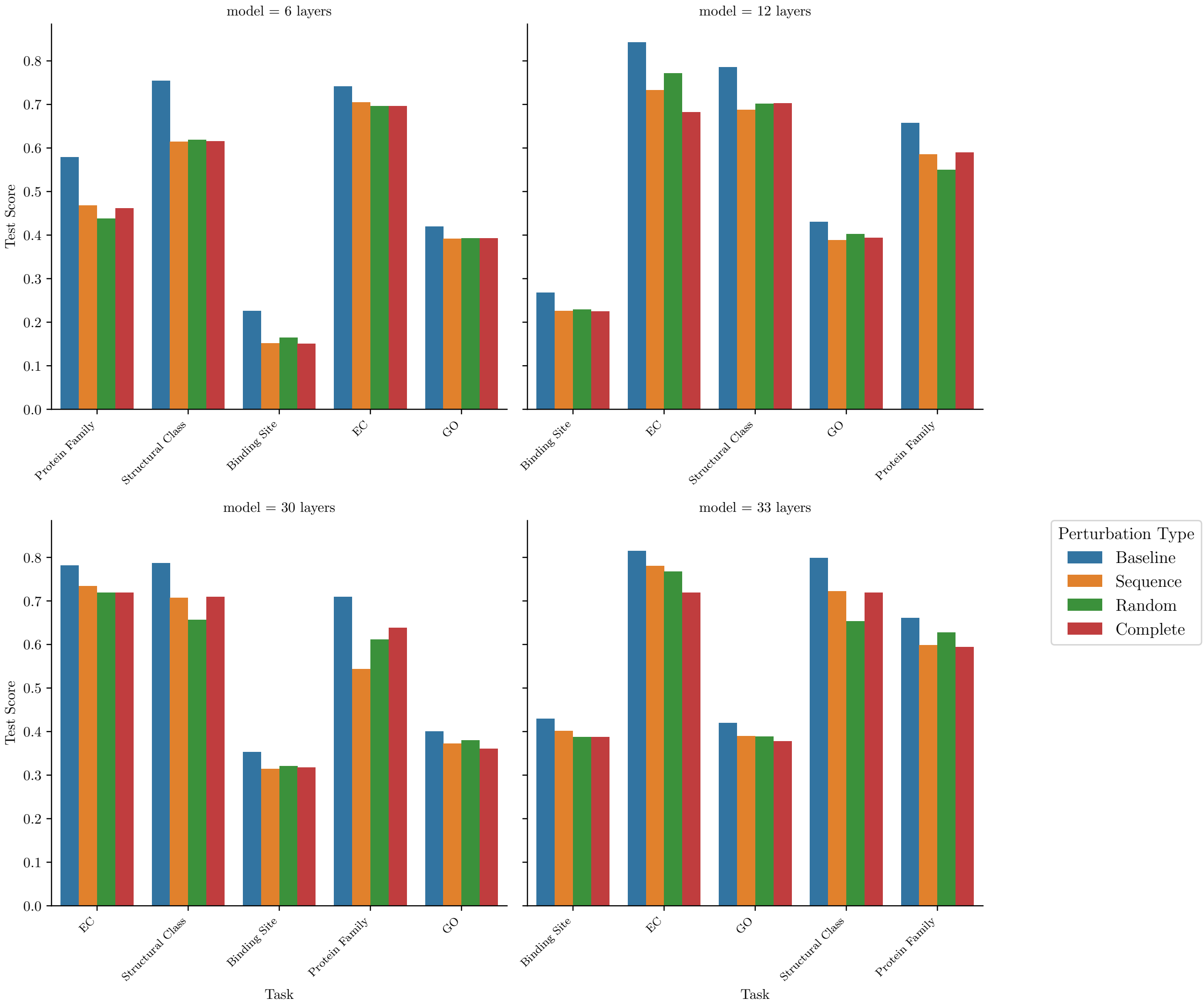

Fig. S4. Effect of perturbations of the underlying graph structure on the performance. The baseline structure is the contact map obtained from the 8Å graph. The sequence graph is the graph that remains when removing all edges not part of adjacent amino acids. The random graph is obtained by swapping edges between nodes while preserving degree distribution. The complete graph is the graph obtained when connecting all nodes together.

Table S4. Comparison of MMSEQS+KNN with PST (fine-tuned and baseline).

Method	EC		GO-BP		GO-MF		GO-CC		Fold Class (ACC)		
	$F_{\max}$	AUPR	$F_{\max}$	AUPR	$F_{\max}$	AUPR	$F_{\max}$	AUPR	Fold	Super.	Fam.
MMSEQS+KNN	0.8458	0.8174	0.5650	0.2966	0.6789	0.5515	0.4962	0.2961	2.785	4.146	83.805
PST (fine-tuned)	0.8970	0.9190	0.4890	0.3480	0.6750	0.6480	0.4750	0.3750	42.300	85.900	99.700
PST	0.8990	0.9180	0.5130	0.3710	0.6860	0.6370	0.5410	0.3870	40.900	83.600	99.400

Table S5. Baseline comparison: MMSEQS+KNN vs. pretrained models. The alignment method only works for protein-level tasks.

Method	GO $F_{\max}$	EC (ACC)	Protein Family (ACC)	Structural Class (ACC)	Binding Site (MCC)	VEP ($\|\rho\|$)
MMSEQS+KNN	0.620	0.796	0.552	0.578	–	–
ESM-2	0.648	0.858	0.698	0.791	0.431	0.489
PST	0.650	0.883	0.704	0.797	0.436	0.501

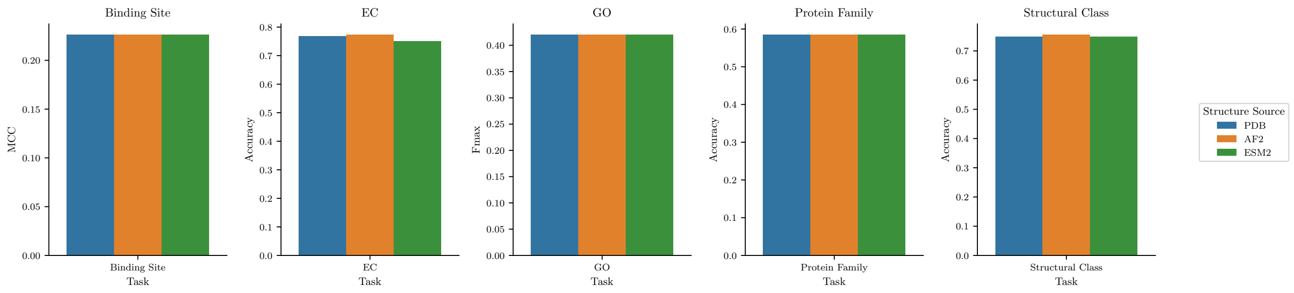

Fig. S5. Performance of PST on various ProteinShake benchmarks when swapping the experimental structure (the baseline, PDB) with either the AlphaFold 2 structure (AF2) or the ESMFold structure (ESM2). Those experiments were conducted with a small PST model with 6 layers which was not fine-tuned on that the specific task.

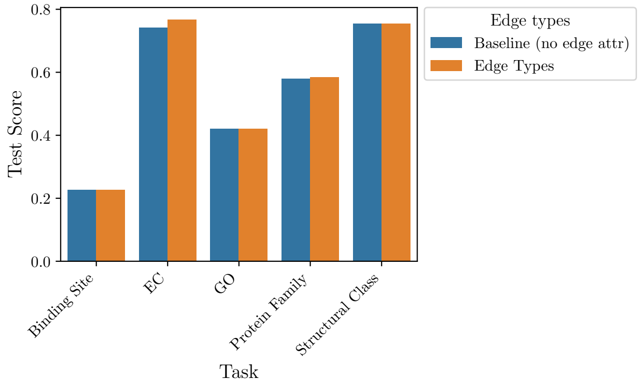

Fig. S6. Performance of PST with and without considering edge types as attributes. The baseline PST does not include any edge attributes. The test scores are the same as those indicated in figure 4 for each task.

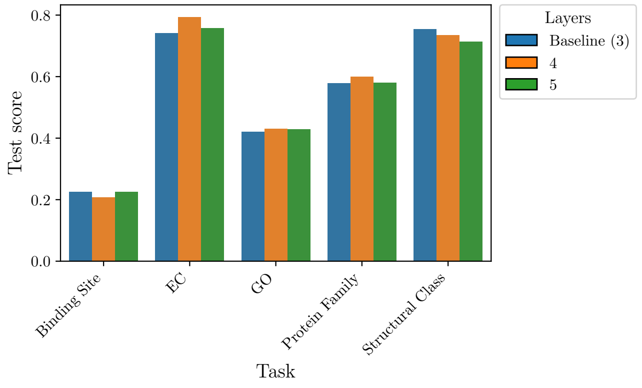

Fig. S7. Performance of PST variants with a varying number of GIN layers. 3 layers are in the baseline PST model. The test scores are the same as those indicated in figure 4 for each task.

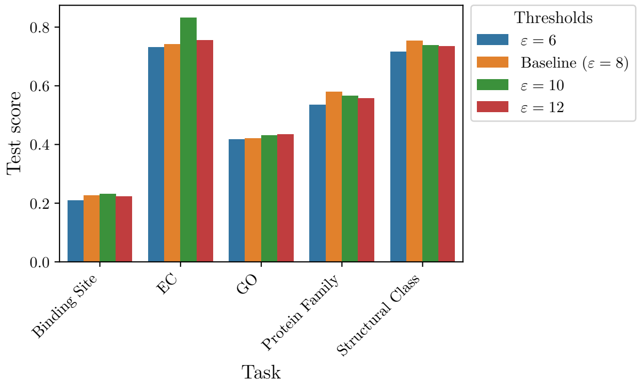

3

Fig. S8. Performance of PST variants with varying numbers of thresholds used for graph construction. The baseline is indicated with a threshold of 8.0 Å. The test scores are the same as those indicated in figure 4 for each task.

References

Bhatnagar, A. *et al.* (2025). Scaling unlocks broader generation and deeper functional understanding of proteins. *bioRxiv*.

Chen, B. *et al.* (2023). xTrimoPGLM: Unified 100B-Scale Pre-trained Transformer for Deciphering the Language of Protein. *bioRxiv*.

Derevyanko, G. *et al.* (2018). Deep convolutional networks for quality assessment of protein folds. *Bioinformatics*, **34**(23), 4046–4053.

Elnaggar, A. *et al.* (2023). Ankh: Optimized Protein Language Model Unlocks General-Purpose Modelling. *arXiv preprint arXiv:2301.06568*.

ESM Team (2024). ESM Cambrian: Revealing the mysteries of proteins with unsupervised learning.

Fowler, D. M. and Fields, S. (2014). Deep mutational scanning: a new style of protein science. *Nature methods*, **11**(8), 801–807.

Gaujac, B. *et al.* (2024). Learning the Language of Protein Structure.

Gligorijević, V. *et al.* (2021). Structure-based protein function prediction using graph convolutional networks. *Nature communications*, **12**(1), 3168.

Hermosilla, P. and Ropinski, T. (2022). Contrastive Representation Learning for 3D Protein Structures. *Pre-print*.

Hermosilla, P. *et al.* (2021). Intrinsic-Extrinsic Convolution and Pooling for Learning on 3D Protein Structures. In *ICLR*.

Hou, J. *et al.* (2018). DeepSF: deep convolutional neural network for mapping protein sequences to folds. *Bioinformatics*, **34**(8), 1295–1303.

Hu, M. *et al.* (2022). Exploring evolution-based &-free protein language models as protein function predictors. In *NeurIPS*.

Ingraham, J. *et al.* (2019). Generative models for graph-based protein design. In *NeurIPS*, volume 32.

Jiao, Y. *et al.* (2023). Struct2GO: protein function prediction based on graph pooling algorithm and AlphaFold2 structure information. *Bioinformatics*, **39**(10), btad637.

Jumper, J. *et al.* (2021). Highly accurate protein structure prediction with AlphaFold. *Nature*, **596**(7873), 583–589.

Lai, L. and Xu, J. (2022). Accurate protein function prediction via graph attention networks with predicted structure information. *Bioinformatics*, **38**(1), 90–97.

Lee, Y. *et al.* (2025). MEGA-GO: functions prediction of diverse protein sequence length using Multi-scalE Graph Adaptive neural network. *Bioinformatics*, **41**(2), btaf032.

Lin, P. *et al.* (2023). Deep transfer learning for inter-chain contact predictions of transmembrane protein complexes. *Nature Communications*, **14**(1), 4935.

Madani, A. *et al.* (2023). Large language models generate functional protein sequences across diverse families. *Nature Biotechnology*, **41**(8), 1099–1106.

Meier, J. *et al.* (2021). Language models enable zero-shot prediction of the effects of mutations on protein function. In *NeurIPS*, volume 34, pages 29287–29303.

Mi, J. *et al.* (2024). GGN-GO: geometric graph networks for predicting protein function by multi-scale structure features. *Briefings in Bioinformatics*, **25**(6), bbae559.

Mohamadi, A. *et al.* (2022). An ensemble 3D deep-learning model to predict protein metal-binding site. *Cell Reports Physical Science*, **3**(9).

Nijkamp, E. *et al.* (2023). ProGen2: Exploring the boundaries of protein language models. *Cell Systems*, **14**(11), 968–978.e3.

Riesselman, A. J. *et al.* (2018). Deep generative models of genetic variation capture the effects of mutations. *Nature methods*, **15**(10), 816–822.

Shanehsazzadeh, A. *et al.* (2020). Is transfer learning necessary for protein landscape prediction? *arXiv preprint arXiv:2011.03443*.

Steinegger, M. and Söding, J. (2017). Mmseqs2 enables sensitive protein sequence searching for the analysis of massive data sets. *Nature biotechnology*, **35**(11), 1026–1028.

Su, J. *et al.* (2024). SaProt: Protein Language Modeling with Structure-aware Vocabulary. In *ICLR*.

Wang, Z. *et al.* (2022). Lm-gvp: an extensible sequence and structure informed deep learning framework for protein property prediction. *Scientific reports*, **12**(1), 6832.

Xu, K. *et al.* (2018). How Powerful are Graph Neural Networks? In *ICLR*.

Yan, X. *et al.* (2022). PointSite: a point cloud segmentation tool for identification of protein ligand binding atoms. *Journal of Chemical Information and Modeling*, **62**(11), 2835–2845.

Zhang, Z. *et al.* (2022). Protein Representation Learning by Geometric Structure Pretraining. In *ICLR*.

Zhang, Z. *et al.* (2023). A systematic study of joint representation learning on protein sequences and structures. *arXiv preprint arXiv:2303.06275*.

Zhao, C. *et al.* (2022). PANDA2: protein function prediction using graph neural networks. *NAR Genomics and Bioinformatics*, **4**(1), lqac004.

Zhao, C. *et al.* (2024). PANDA-3D: protein function prediction based on AlphaFold models. *NAR Genomics and Bioinformatics*, **6**(3), lqae094.

Zheng, Z. *et al.* (2023). Structure-informed language models are protein designers. In *ICML*.
